# Comment on Cerland, L. et al. Incidence and Consequences of Near-Drowning-Related Pneumonia—A Descriptive Series from Martinique, French West Indies. *Int. J. Environ. Res. Public Health* 2017, *14*, 1402

**DOI:** 10.3390/ijerph15040706

**Published:** 2018-04-10

**Authors:** Ana Catarina Queiroga, Jonathon Webber, Andrew C. Schmidt, Justin R. Sempsrott, Roberto Barcala-Furelos, Michael Tipton, David Szpilman

**Affiliations:** 1 EPI-Unit, Institute of Public Health, University of Porto, 4050-600 Porto, Portugal; 2 Department of Anaesthesiology, The University of Auckland, Auckland 1142, New Zealand; jweb018@aucklanduni.ac.nz; 3 Surf Life Saving New Zealand, P.O. Box 39129, Wellington Mail Centre, Lower Hutt 5045, New Zealand; 4 Department of Emergency Medicine, University of Florida College of Medicine-Jacksonville, Jacksonville, FL 32209, USA; andrew.schmidt@jax.ufl.edu; 5 Lifeguards without Borders, P.O. Box 737, Kuna, ID 83634, USA; justin@lifeguardswithoutborders.org; 6 REMOSS Research Group, Lifesaving and Motor Skill, Faculty of Education and Sport Sciences, University of Vigo, 36005 Pontevedra, Spain; roberto.barcala@uvigo.es; 7 Extreme Environments Laboratory, Department of Sport & Exercise Science, University of Portsmouth, Portsmouth PO1 2ER, UK; michael.tipton@port.ac.uk; 8 Sociedade Brasileira de Salvamento Aquático, Barra da Tijuca, Rio de Janeiro RJ 22631-004, Brazil; david@szpilman.com

We read with great interest the recent paper by Cerland et al. on the frequency, nature, and consequences of post-drowning pneumonia [1]. We applaud the authors for raising awareness of drowning and the management of non-fatal drowning patients, particularly in the context of antibiotic stewardship. Our concern, and reason for corresponding, is the continued use of outdated terminology in the title and text of this article, namely the term ‘near drowning’.

In 2003, an advisory statement of the International Liaison Committee on Resuscitation (ILCOR) recommending the use of a uniform way of reporting data on drowning was published [2], with an update in 2016 [3]. This consensus-based document, inspired by the Utstein style of data collection for cardiac arrest, was created to provide more consistency in describing drowning research and improve comparability between individual studies. In this statement, drowning was defined as ‘a process resulting in primary respiratory impairment from submersion/immersion in a liquid medium. Implicit in this definition is that a liquid/air interface is present at the entrance of the victim’s airway, preventing the victim from breathing air. The victim may live or die after this process, but whatever the outcome, he or she has been involved in a drowning incident’ [3]. The use of the term ‘near-drowning’, which was considered to be confusing, was thus abandoned [3]. For more than a decade, this uniform definition of ‘drowning’ has been adopted by the World Health Organisation (WHO) and it has also been incorporated in the European Resuscitation Council guidelines [4,5]. The details of the inception of this definition and accepted terminology is well-described in the WHO Bulletin by van Beeck et al. referenced by the authors of this current paper.

We believe that the use of uniform terminology describing drowning incidents is vital for good, qualitative comparisons of drowning research, and improving patient outcomes. We therefore urge all authors to describe drowning incidents using the terminology advised by ILCOR and the WHO. Those who survive the initial incident should be considered to have survived a non-fatal drowning.
